# Supplementary material for: Temporal Trends of Acute Hepatitis A in Brazil and Its Regions
Source: Viruses. 2022 Dec 8;14(12):2737. doi: 10.3390/v14122737 (PMC9784953; doi:10.3390/v14122737)
Supplement: Supplementary file 1 [file viruses-14-02737-s001.zip › viruses-2010279-supplementary.pdf]

Supplementary material Table S1. Results of temporal trends analysis, AAPC and APC, with identified time periods, by region, gender and age groups.
*results that achieved statistical significance (p<0.05)

Age groups	Gender	Age Groups	AAPC	APC
North	Females	Less than 5	-35.0*, -44.1 to -24.3	2007 to 2014: -4.3, -19.4 to 13.6<br>2014 to 2018: -66.9*, -78.0 to -50.4
		5 to 19	-28.6*, -39.9 to -15.1	2007 to 2014:-0.4, -18.0 to 21.1<br>2014 to 2018:-60.1*, -74.8 to -36.7
		20 to 39	-17.9*, -25.5 to -9.5	2007 to 2014:-0.2, -10.6 to 11.4<br>2014 to 2018:-41.7*, -55.0 to -24.4
		Above 40	-10.6*, -16.0 to -4.7	2007 to 2018:-10.6*, -16.0 to -4.7
	Males	Less than 5	-40.1*, -45.3 to -34.4	2007 to 2015: -10.2*, -16.8 to -3.2<br>2015 to 2018: -79.6*, -85.6 to -71.2
		5 to 19	-29.8*, -39.7 to -18.3	2007 to 2014: 3.2, -13.1 to 22.4<br>2014 to 2018: -64.2*, -76.1 to -46.3
		20 to 39	-19.7*, -29.3 to -8.8	2007 to 2014: 2.1, -11.5 to 17.9<br>2014 to 2018: -47.3*, -62.5 to -26.0
		Above 40	-0.8, -12.0 to 11.8	2007 to 2013: -0.4, -8.5 to 8.3<br>2013 to 2016: -23.1, -53.3 to 26.5<br>2016 to 2018: 43.7, -12.7 to 136.5
Northeast	Females	Less than 5	-41.7*, -58.9 to -17.3	2007 to 2014:-19.3, -42.0 to 12.3<br>2014 to 2017:-72.7*, -92.1 to -6.0
		5 to 19	-38.6*, -43.0 to -34.0	2007 to 2014:-17.1*, -23.6 to -10.0<br>2014 to 2018:-63.8*, -70.2 to -56.0
		20 to 39	-22.5*, -29.3 to -15.1	2007 to 2014:-13.5*, -21.9 to -4.1<br>2014 to 2018:-36.1*, -49.9 to -18.5
		Above 40	-7.0, -15.4 to 2.2	2007 to 2009:15.7, -35.2 to 106.6<br>2009 to 2018:-11.4*, -16.0 to -6.6
	Males	Less than 5	-40.4*, -43.5 to -37.0	2007 to 2014:-18.0*, -22.9 to -12.7<br>2014 to 2018:-65.9*, -70.7 to -60.3
		5 to 19	-41.2*, -47.9 to -33.7	2007 to 2014:-18.0*, -28.3 to -6.1<br>2014 to 2018:-67.2*, -76.2 to -54.9
		20 to 39	-26.2*, -32.9 to -18.8	2007 to 2014:-15.8*, -24.4 to -6.2<br>2014 to 2018:-41.3*, -54.5 to -24.3
		Above 40	-7.4*, -9.8 to -5.0	2007 to 2018:-7.4*, -9.8 to -5.0

Central-West	Females	Less than 5	-33.0*, -40.9 to -24.1	2007 to 2014: -19.9*, -30.9 to -7.1<br>2014 to 2018: -51.0*, -66.2 to -29.1
		5 to 19	-33.2*, -46.9 to -16.0	2007 to 2014: -15.6*, -28.4 to -0.6<br>2014 to 2018: -70.4, -91.3 to 0.7
		20 to 39	-19.7*, -26.1 to -12.7	2007 to 2018: -19.7*, -26.1 to -12.7
		Above 40	-6.1*, -10.1 to -1.9	2007 to 2018: -6.1*, -10.1 to -1.9
	Males	Less than 5	-36.7*, -46.3 to -25.4	2007 to 2014: -20.3*, -33.8 to -4.1<br>2014 to 2018: -57.7*, -72.7 to -34.5
		5 to 19	-41.2*, -53.0 to -26.4	2007 to 2014: -19.2, -37.3 to 4.1<br>2014 to 2018: -66.3*, -81.5 to -38.6
		20 to 39	-17.6*, -21.9 to -13.0	2007 to 2018: -17.6*, -21.9 to -13.0
		Above 40	-4.3*, -8.4 to -0.1	2007 to 2018: -4.3*, -8.4 to -0.1
South	Females	Less than 5	-34.9*, -42.4 to -26.3	2007 to 2018: -34.9*, -42.4 to -26.3
		5 to 19	-39.5*, -43.6 to -35.1	2007 to 2018 -39.5*, -43.6 to -35.1
		20 to 39	-15.3*, -23.0 to -6.8	2007 to 2010: -11.7, -32.0 to 14.8<br>2010 to 2016: -25.4*, -33.6 to -16.1<br>2016 to 2018: 16.2, -31.2 to 96.3
		Above 40	-3.0, -6.5 to 0.6	2007 to 2018: -3.0, -6.5 to 0.6
	Males	Less then 5	-34.6*, -38.8 to -30.2	2007 to 2018: -34.6*, -38.8 to -30.2
		5 to 19	-35.7*, -38.4 to -32.9	2007 to 2018: -35.7*, -38.4 to -32.9
		20 to 39	-6.4, -16.0 to 4.2	2007 to 2016: -19.6*, -24.4 to -14.6<br>2016 to 2018: 85.9, -4.1 to 260.3
		Above 40	-1.7, -4.2 to 0.9	2007 to 2018: -1.7, -4.2 to 0.9
Southeast	Females	Less than 5	-33.0*, -43.0 to -21.2	2007 to 2014: -20.9*, -34.3 to -4.9<br>2014 to 2018: -49.9*, -68.1 to -21.2
		5 to 19	-30.1*, -38.6 to -20.3	2007 to 2018: -30.1*, -38.6 to -20.3
		20 to 39	-2.0, -11.3 to 8.4	2007 to 2016: -12.7*, -17.4 to -7.6<br>2016 to 2018: 64.8, -11.0 to 205.0

	Above 40	2.4, -5.0 to 10.3	2007 to 2009: -16.7, -47.4 to 31.8<br>2009 to 2018: 7.2*, 2.8 to 11.7
Males	Less than 5	-29.6*, -41.5 to -15.2	2007 to 2014: -19.8*, -34.9 to -1.1<br>2014 to 2018: -43.9*, -65.8 to -8.0
	5 to 19	-27.6*, -34.8 to -19.6	2007 to 2018: -27.6*, -34.8 to -19.6
	20 to 39	11.0, -1.6 to 25.3	2007 to 2016: -11.3*, -17.1 to -5.1<br>2016 to 2018: 205.2*, 45.8 to 539.0
	Above 40	9.9*, 1.0 to 19.7	2007 to 2016: 2.8, -2.0 to 7.8<br>2016 to 2018: 48.9, -11.6 to 150.7
